# Survival of Esophageal Cancer in China: A Pooled Analysis on Hospital-Based Studies From 2000 to 2018

**DOI:** 10.3389/fonc.2019.00548

**Published:** 2019-06-27

**Authors:** Haifeng Hou, Zixiu Meng, Xuan Zhao, Guoyong Ding, Ming Sun, Wei Wang, Youxin Wang

**Affiliations:** ^1^School of Public Health, Taishan Medical University, Taian, China; ^2^Beijing Key Laboratory of Clinical Epidemiology, School of Public Health, Capital Medical University, Beijing, China; ^3^School of Medical and Health Sciences, Edith Cowan University, Perth, WA, Australia

**Keywords:** esophageal cancer, survival, meta-analysis, hospital-based study, China

## Abstract

**Background:** Esophageal cancer (EC) causes more than 400 thousand deaths per year, and half of them occur in China. There are discrepancies regarding the survival of EC patients between population-based surveillance studies and hospital-based studies.

**Objectives:** We aimed to synthesize the survival data from hospital-based EC studies in the Chinese population from 2000 to 2018 and to compare the survival rates between EC patients with different clinical classifications.

**Methods:** The protocol of this systematic review was registered in PROSPERO (CRD-42019121559). We searched Embase, PubMed, CNKI, and Wanfang databases for studies published between January 1, 2000 and December 31, 2018. We calculated the pooled survival rates and 95% confidence intervals (CIs) by Stata software (V14.0).

**Results:** Our literature search identified 933 studies, of which 331 studies with 79,777 EC patients met the inclusion criteria and were included in meta-analyses. The pooled survival rates were 74.1% (95% CI: 72.6–75.7%) for 1-year survival, 49.0% (95% CI: 44.2–53.8%) for 2-years survival, 46.0% (95% CI: 42.6–49.5%) for 3-years survival, and 40.1% (95% CI: 33.7–46.4%) for 5-years survival. An increased tendency toward EC survival was verified from 2000 to 2018. In addition, discrepancies were observed between EC patients with different clinical classifications (e.g., stages, histologic types, and cancer sites).

**Conclusions:** Our findings showed a higher survival rate in hospital-based studies than population-based surveillance studies. Although this hospital-based study is subject to potential representability and publication bias, it offers insight into the prognosis of patients with EC in China.

## Introduction

Esophageal cancer (EC) is among the top causes of cancer-related mortality globally, resulting in more than 400 thousand deaths each year (1). Because its incidence is increasing, EC remains a global concern, especially in under-developed regions (2). Annually, more than half of new cases are diagnosed in China, where a so-called EC belt permanently exists around Taihang Mountain (3, 4). EC ranks fifth in incidence (21.17 per 100,000), and fourth in mortality (15.58 per 100,000) among all malignant tumors in China (5, 6). Carcinogen exposure (e.g., nitrosamines and their precursors, fungi, trace minerals, and polycyclic hydrocarbons), nutritional deficiency, lifestyles, and genetics contribute to the multistage development of EC through (1) localized injury, (2) inflammation, (3) mutagenesis, and (4) carcinogenesis (7, 8).

Although strategies for preventing EC are necessary, measures to reduce morbidity or improve survival are also extremely important. Therefore, a national screening program was launched in 2005 in areas in China with a high prevalence of EC, especially rural regions (9). This program includes endoscopic examination for high-risk individuals and early surgery in the treatment of patients with EC (5). Several studies have reported a 50% decrease in EC deaths among subjects served in this healthcare program (9, 10). Also, a nationwide population-based cancer registry system was initiated to collect cancer data across China in 2002 (11, 12). These population-based survival databases enable both temporal and spatial surveillance of the overall state of different cancers (13). However, incomplete follow-up for registered individuals can bias survival estimates in population-based surveillance studies. When the registered data do not cover all eligible cancer deaths, the survival statistics might be overestimated, particularly for cancers with high fatality rates (14). Meanwhile, many clinical investigations have reported the prognosis of patients with cancer within hospitals according to clinical research criteria. These hospital-based survival investigations can reflect comprehensive treatment effectiveness with relatively lower loss to follow-up than population-based studies (15). Discrepancies exist regarding the survival of EC patients between reports from the national cancer registry system and hospital-based studies, as well as between hospital-based studies. The present study aimed to systematically synthesize all eligible survival data reported in hospital-based clinical studies in the Chinese population in order to estimate the prognosis of hospitalized patients with EC.

## Materials and Methods

The systematic review and meta-analyses were conducted in accordance with the criteria of the Preferred Reporting Items for Systematic Reviews and Meta-Analyses (PRISMA) (16). The reported PRISMA Checklist is available in the online Table S1. As a systematic review, the protocol was registered in PROSPERO (No. CRD-42019121559, https://www.crd.york.ac.uk/PROSPERO/).

### Literature Search Strategy

We searched Embase, PubMed, and Web of Science for English language literature, as well as CNKI and Wanfang for Chinese literature published between January 1, 2000 and December 31, 2018. The combination of “China,” “Chinese,” “survival,” “hospital,” “esophageal cancer,” “carcinoma of esophagus,” “esophageal carcinoma,” “esophagus cancer,” “cancer of esophagus,” “esophageal cancer,” “carcinoma of esophagus,” “esophageal carcinoma,” “esophagus cancer,” and “cancer of esophagus” in English or Chinese were used in the literature search. References cited in the included articles were further reviewed. The detailed search strategy is shown in Table S2.

### Selection Criteria and Quality Assessment

First, two authors (XZ and ZM) independently reviewed the titles and abstracts of the retrieved publications. Second, the full text and online supplementary data were read to determine the eligibility and quality of the literature (Table S3). Any uncertainties and discrepancies were resolved by the third investigator (HH) through discussion. The inclusion criteria were (1) EC was diagnosed by pathological examination; (2) the following data were available: number of included EC patients, number of survived cases, or survival rates of EC patients; (3) the survival data were obtained from hospital-based studies that assessed the prognosis of hospitalized inpatients; (4) the ethnicity of all EC patients was Chinese. For studies reporting on both Chinese and other populations, only the data for the Chinese population were included. The exclusion criteria were as follows: (1) *in vitro* studies or animal studies; (2) reviews; (3) studies that did not refer to a Chinese population; (4) studies without survival data; (5) studies on community-based populations; (6) low-quality studies.

### Data Extraction

The following data were extracted from included studies: (1) first author's name, publication year, characteristics of EC patients (e.g., age, gender, region, cancer site, clinical stage, and clinical type), survival rate or number of survival cases, and study design. If data of a specific population were reported in several studies or published more than once, the most recently published or the largest sampled study was included. Data in each subgroup (study design, gender, regions, cancer site, clinical type, and stage) were also extracted for subgroup analyses.

### Statistical Analysis

The pooled survival rate and 95% confidence interval (CI) of EC were calculated by Stata V14.0 (Stata Corp, College Station, TX, USA). Cochran's *Q*-test for heterogeneity, complemented by the *I*^2^ statistic, was implemented to measure the level of heterogeneity across original studies. A *P* > 0.10 and *I*^2^ < 50% indicated no significant heterogeneity, and a fixed effect model was utilized to calculate the pooled survival rate. Otherwise, significant heterogeneity was assumed, then the random effect model was carried out subsequently by the DerSimonian-Laird method (17). Subgroup analyses on gender, cancer site, clinical type, and stage, temporal trend, and study design were conducted, respectively. To assess the strength and stability of pooled results, sensitivity analyses were also conducted by successive removal of an eligible study each time. The publication bias was estimated by funnel plot asymmetry and Begg's adjusted rank correlation test. A *P-*value < 0.05 was considered to represent statistical significance.

## Results

### Systematic Review and Eligible Studies

**Figure 1:**
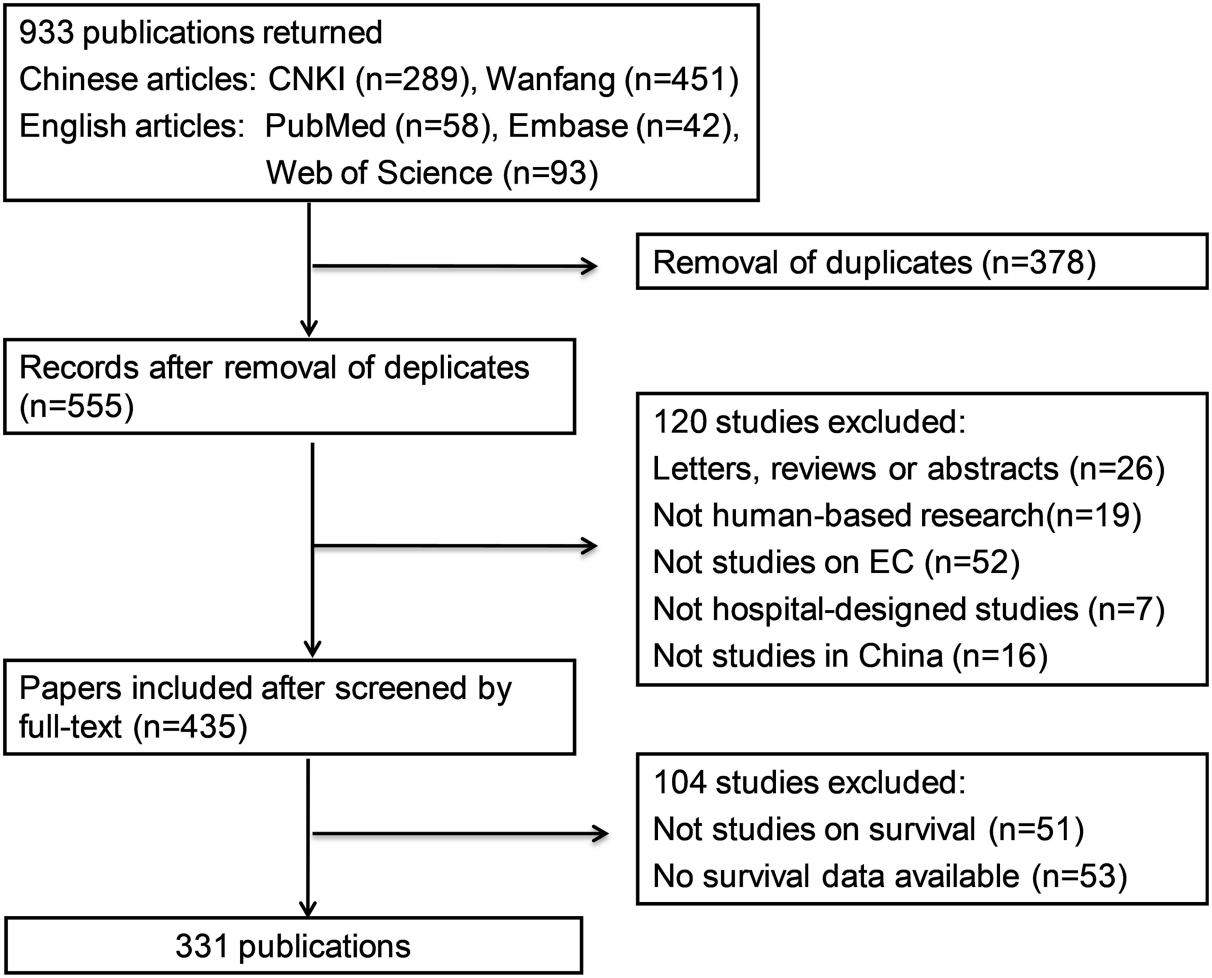
Flow diagram of literature screening.

**Figure 2:**
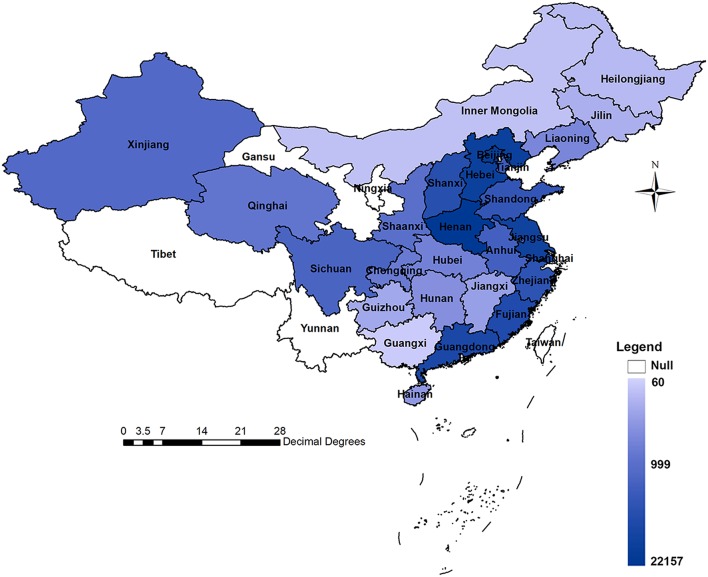
Distribution of included studies.

Our literature search identified 933 publications, of which 289 were from CNKI, 451 were from Wanfang, 42 were from Embase, 58 were from PubMed, and 93 were from Web of Science. Two authors (ZM and ZX) reviewed the retrieved literature independently and then excluded 378 reduplicate publications. Among the 555 publications involved in the abstract and full-text review, 26 were not original studies, 19 were not human-based research, 52 were not in reference to EC, seven were not designed as hospital-based studies, 16 were not conducted among a Chinese population, 51 were not investigations on survival or prognosis, and 53 did not provide appropriate survival data. Finally, 331 publications with 79,777 EC patients (52,273 males/21,874 females/5,630 unavailable; mean age 51.8 years) that met the predefined criteria were included in our study. The flow diagram for screening articles is shown in Figure 1. The distributions of the studies included are shown in Figure 2 and additional details are provided in Table S4. There were 104 prospective studies and 227 retrospective studies. A total of 118 studies reported survival of patients with esophageal squamous cell carcinoma (ESCC), eight reported survival of patients with esophageal small cell carcinoma (SCC), two reported survival of patients with esophageal adenocarcinoma (EAC), 119 reported the combined data of more than one histologic type, and 84 studies did not mention the histologic classifications. Of the 331 studies, 210 were conducted in the high prevalence areas of China (e.g., Fujian, Guangdong, Henan, Jiangsu, Shandong, Shanxi, Shaanxi, and Sichuan provinces).

### Pooled Results of EC Survival

As shown in Table 1, the observed survival rates in all eligible studies were synthesized by a random effect model. The overall pooled results were 74.1% (95% CI: 72.6–75.7%) for 1-year survival, 49.0% (95% CI: 44.2–53.8%) for 2-years survival, 46.0% (95% CI: 42.6–49.5%) for 3-years survival, and 40.1% (95% CI: 33.7–46.4%) for 5-years survival.

**Table 1:** Pooled survival and 95% CI of esophageal cancer in low and high prevalence areas.

**Survival**	**Low prevalence areas**		**High prevalence areas**		**Overall**	
	***N***	**SR (%)**	***N***	**SR (%)**	***N***	**SR (%)**
1-year	115	74.1 (72.0–76.3)	117	74.2 (71.8–76.5)	232	74.1 (72.6–75.7)
2-years	62	47.6 (40.2–55.0)^a^	63	50.4 (45.0–55.8)^a^	125	49.0 (44.2–53.8)^a^
3-years	107	45.8 (40.2–51.5)^a^	104	46.2 (42.5–49.9)^a^	211	46.0 (42.6–49.5)^a^
5-years	47	41.3 (33.0–49.6)^a^	26	37.1 (29.3–45.0)^ab^	73	40.1 (33.7–46.4)^a^

SR, survival rate; CI, confidence interval; N, number of included studies; Between-group comparisons:

a P < 0.05 compared with 1-year survival;

b *P < 0.05 compared with 2-years survival*.

**Figure 3:**
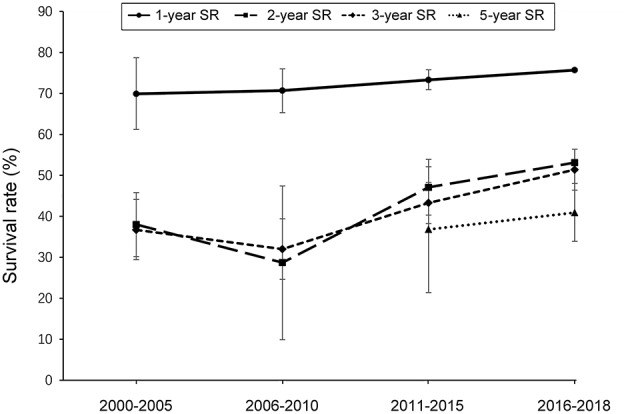
Temporal trends of survival of esophageal cancer from 2000 to 2018. Between-group comparisons: the 1-year survival rates are significantly higher than 2-, 3-, and 5-years survival rates between 2000 and 2018 (*P* < 0.05).

To examine the survival profiles from 2000 to 2018, we conducted analyses on time-trend survival of patients with EC. As shown in Figure 3 and Table S5, a tendency for increased survival was evidenced by the pooled survival rates from 2006 to 2018. The same trends were found in both men and women (Tables S6, S7), except that the 5-years survival rates among men decreased from 2006 to 2015.

**Figure 4:**
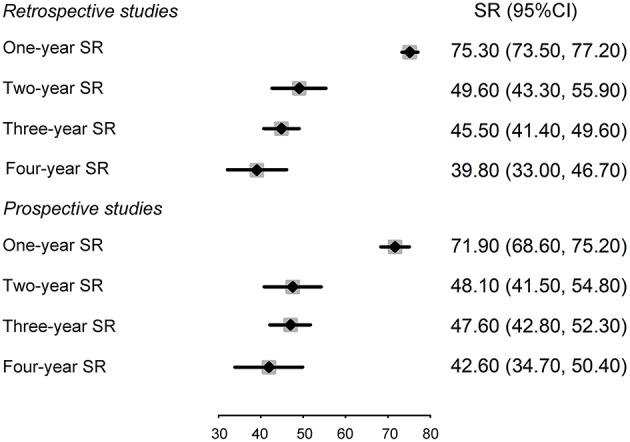
Forest plot of meta-analysis of retrospective and prospective studies.

Subgroup analysis on study design (Figure 4 and Table S8) indicated that the pooled survival rates in retrospective studies were 75.3% (95% CI: 73.5–77.2%) for 1-year survival, 49.6% (95% CI: 43.3–55.9%) for 2-years survival, 45.5% (95% CI: 41.4–49.6%) for 3-years survival, and 39.8% (95% CI: 33.0–46.7%) for 5-years survival. Meanwhile, the pooled survival rates in prospective studies were 71.9% (95% CI: 68.6–75.2%) for 1-year survival, 48.1% (95% CI: 41.5–54.8%) for 2-years survival, 47.6% (95% CI: 42.8–52.3%) for 3-years survival, and 42.6% (95% CI: 34.7–50.4%) for 5-years survival. Moreover, subgroup analyses by sex showed higher rates of 2-, 3-, and 5-years survival among women than for those of men, while the differences were not statistically significant (Tables S6, S7).

We also synthesized survival rates in the high prevalence areas of China (e.g., Fujian, Guangdong, Henan, Jiangsu, Shandong, Shanxi, Shaanxi, and Sichuan provinces), where the incidence rates of EC are higher than other provinces. The pooled survival rates in these high prevalence areas were 74.2% (95% CI: 71.8–76.5%) for 1-year survival, 50.4% (95% CI: 45–55.8%) for 2-years survival, 46.2% (95% CI: 42.5–49.9%) for 3-years survival, and 37.1% (95% CI: 29.3–45.0%) for 5-years survival (Table 1). The prognosis of EC in high prevalence areas was similar with low prevalence areas.

### Subgroup Analyses of EC Survival by Clinical Classifications

Thirty-five studies that involved 13,055 patients reported the TNM stage of ED. As shown in Table 2, the pooled analyses showed a decreasing linear trend of survival (including 1-, 2-, 3-, and 5-years survival) for patients with T1, T2, T3, and T4 stage EC. A decreased trend was found in accordance with the TNM stage classifications. The 5-years survival of patients with stage T4 EC was 25.4% (95% CI: 14.9–36.0%), which was significantly lower than the survival for patients with stage T1 EC (75.2%, 95% CI: 68.4–82.0%).

**Table 2:** Pooled survival and 95% CI of esophageal cancer in different clinical stages.

**Categories**	**Stage**	***N***	**SR (%)**
1-year survival	T1	6	95.6 (93.5–97.7)
	T2	8	84.7 (79.3–90.0)^a^
	T3	12	74.6 (68.8–80.4)^a^
	T4	17	52.8 (39.9–65.7)^abc^
	Overall	43	71.7 (66.5–76.9)
2-years survival	T1	3	87.0 (71.0–103.0)
	T2	3	51.4 (39.9–62.9)^a^
	T3	4	34.5 (28.8–40.2)^a^
	T4	2	11.4 (2.2–25.1)^abc^
	Overall	12	41.8 (13.7–69.8)
3-years survival	T1	10	81.0 (75.5–86.4)
	T2	13	61.5 (53.6–69.3)^a^
	T3	17	45.0 (36.9–53.1)^ab^
	T4	11	36.3 (21.8–50.7)^ab^
	Overall	51	53.3 (46.5–60.1)
5-years survival	T1	13	75.2 (68.4–82.0)
	T2	14	51.6 (43.3–59.9)^a^
	T3	16	38.5 (30.6–46.4)^a^
	T4	10	25.4 (14.9–36.0)^ab^
	Overall	53	48.4 (41.6–55.1)

SR, survival rate; CI, confidence interval; N, number of included studies. Between-group comparisons:

a P < 0.05 compared with T1 group;

b P < 0.05 compared with T2 group;

c *P < 0.05 compared with T3 group*.

The major histologic type of EC in China is ESCC, which accounts for 90% of new cases, while adenocarcinoma is more common in Western countries (18–20). The pooled results (Table 3) show that the 5-years survival rate of SCC (15.5%, 95% CI: 12.4–18.6%) was significantly lower than that of ESCC (41.7%, 95% CI: 32.4–51.0%). The 1- and 3-years survival rates of SCC were significantly lower as well. However, only two studies reported survival of EAC, for which the pooled 1- and 3-years survival rates were 80.9% (95% CI: 62.3–99.5%) and 45.7 (95% CI: 23.2–68.2%), respectively.

**Table 3:** Pooled survival and 95% CI of esophageal cancer of different histologic types.

**Categories**	**Types**	***N***	**SR (%)**
1-year survival	Combined	145	74.1 (72.1–76.0)
	ESCC	80	75.3 (72.7–78.0)
	SCC	5	55.6 (40.4–70.8)^a^
	EAC	2	80.9 (62.3–99.5)
	Overall	232	74.1 (72.6–75.7)
2-years survival	Combined	79	50.40 (44.5–56.2)
	ESCC	41	48.70 (39.9–57.6)
	SCC	4	24.40 (4.6–44.2)
	EAC	1	–
	Overall	125	49.00 (44.2–53.8)
3-years survival	Combined	127	47.8 (43.2–52.3)
	ESCC	78	44.6 (39.4–49.7)
	SCC	4	18.8 (2.6–35.0)^a^
	EAC	2	45.7 (23.2–68.2)
	Overall	210	46.0 (42.6–49.5)
5-years survival	Combined	37	41.30 (31.7–50.9)
	ESCC	31	41.70 (32.4–51.0)
	SCC	4	15.50 (12.4–18.6)^a^
	EAC	1	–
	Overall	72	40.10 (33.7–46.4)

SR, survival rate; CI, confidence interval; N, number of included studies; ESCC, esophageal squamous cell carcinoma; SCC, esophageal small cell carcinoma; EAC, esophageal adenocarcinoma; Combined, esophageal cancer patients not classified by histology; N, number of included original studies. Between-group comparisons:

a *P < 0.05 compared with ESCC group*.

We performed a subgroup meta-analysis on four sites of the original tumors. As shown in Table 4, 1-, 3-, and 5-years survival rates were lower among patients with hypopharynx and cervical EC than patients with thoracic EC.

**Table 4:** The pooled survival and 95% CI of esophageal cancer at different sites.

**Categories**	**Sites**	***N***	**SR (%)**
1-year survival	Hypopharynx and cervical	6	76.5 (68.7–84.4)
	Upper thoracic	15	79.3 (73.7–84.8)
	Mid thoracic	19	77.6 (72.1–83.0)
	Lower thoracic	15	73.3 (66.2–80.3)
	Overall	55	77.1 (73.9–80.3)
2-years survival	Hypopharynx and cervical	2	69.4 (45.0–93.7)
	Upper thoracic	3	49.1 (25.9–72.3)
	Mid thoracic	8	44.5 (35.4–53.6)
	Lower thoracic	5	34.9 (6.4–63.5)
	Overall	18	44.7 (36.2–53.1)
3-years survival	Hypopharynx and cervical	9	40.4 (30.2–50.6)
	Upper thoracic	16	48.1 (42.3–54.0)
	Mid thoracic	20	44.5 (35.2–53.8)
	Lower thoracic	16	46.8 (39.7–53.9)
	Overall	61	45.6 (41.0–50.1)
5-years survival	Hypopharynx and cervical	3	10.7 (0.1–21.4)
	Upper thoracic	11	32.6 (24.8–40.4)^a^
	Mid thoracic	12	37.4 (24.9–49.9)^a^
	Lower thoracic	12	39.6 (31.0–48.1)^a^
	Overall	38	35.2 (28.8–41.5)^a^

SR, survival rate; CI, confidence interval; N, number of included original studies. Between-group comparisons:

a *P < 0.05 compared with hypopharynx group*.

### Results of Publication Bias and Sensitivity Analyses

**Figure 5:**
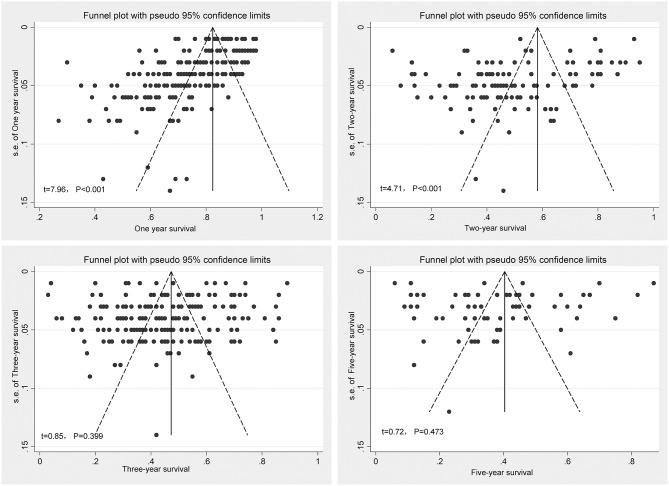
Funnel plots of publication bias analyses.

As shown in Figure 5, the funnel plot analyses and Begg's tests indicated no publication bias in the meta-analyses of 3- and 5-years survival. However, significant publication bias was detected in both 1- and 2-years survival analyses.

To address the strength and stability of the pooled results, we conducted sensitivity analysis by omitting one study at a time. The estimates of pooled survival were not significantly affected by any individual study that was included in our meta-analyses (see Figures S1–S4), indicating that our results had relative strength and stability.

## Discussion

Our study examined the hospital-based survival across China of patients with EC. Among the combined 79,777 patients from 331 hospital-based studies, the overall 5-years survival rate of EC was 40.1%. Tendency analyses show that survival rates of EC have been increasing from 2000 to 2018. Subgroup analyses illustrate that men with EC have poorer survival compared to women.

In China, the regions with a high prevalence of EC are located in Fujian, Guangdong, Henan, Jiangsu, Shandong, Shanxi, Shaanxi, and Sichuan provinces (21–25). We found that EC survival rates in high prevalence areas were similar to those in other regions. It is notable that 90% of ECs in the Chinese population are ESCCs, which is inconsistent with other ethnicities. Our study found that survival of ESCC was significantly higher than SSC in China. As a minor histologic type of EC in China, EAC accounts for <1% of EC (19). Among the hundreds of studies we included, only two reported an independent result on EAC. Due to the lack of such studies, the pooled survival of EAC might not be representative. Moreover, TNM stage is also a crucial determinant for EC survival (26). Our findings showed a decreased trend of survival for patients with T1, T2, T3, and T4 stage EC. The 5-years survival rate of patients with stage T4 EC was nearly three times lower than that of patients with T1 EC. About half of ECs locate in the middle thoracic esophagus, followed by lower thoracic, higher thoracic, and hypopharyngeal parts of the esophagus (27). Our subgroup analyses showed lower survival rates among patients with hypopharynx and cervical EC, except for 2-years survival rates that were obtained from two published studies. These findings verify the relatively poorer prognosis of hypopharynx and cervical EC.

Because China has the highest burden of EC worldwide, our focus is to investigate the key measures of early detection and appropriate treatment for EC patients, as well as the strategies that can be taken to evaluate both the efficiency of preventive policies and the effectiveness of healthcare services across the country (28, 29). The national cancer registration system initiated in 2002 has been reinforced since 2015 and involved 416 population-based sites as of 2016 (30). Although this registry provides information on nationwide epidemics and burdens, it does not include all cancer patients in China. The comparability and validity of the information might vary between sites due to discrepancies in quality controls, as well as temporal heterogeneities. Moreover, follow-up is not easy to implement in the populations served by cancer registries. For instance, it has been reported that the follow-up rate was only between 65 and 71% at some sites (31).

Therefore, hospital-based studies are essential in order to predict the prognosis of patients with EC. We conducted this systematic review to combine the hospital-based survival data of patients with EC that were collected in accordance with clinical research criteria. We supposed that these results presented survival information from the viewpoint of clinical oncologists. Our findings verified an increased tendency toward EC survival from 2000 to 2018. The increase in EC survival has been attributed to improved access to primary health care, greater availability of diagnostic facilities, and improved effectiveness of clinical treatment. The health insurance system of China has been updated to cover all populations since 2003. Access to this insurance support has improved the survival of patients with EC across the country (11).

A large-scale study involving 63,506 patients with EC from 17 population-based cancer registries reported increased survival from 20.9 to 30.3% between 2003 and 2015 (12). However, we found a higher survival rate among the hospital-based population. This discrepancy is because population-based data are collected from non-inclusive study participants, while hospital-based data come from outpatients and inpatients who generally receive more effective therapy. Another population-based study estimated a decrease in survival from 19.1 to 18.1% in urban China (11), in which EC was the only cancer with a lower survival rate in urban compared to rural areas. Such results might be due to observation bias, which results from loss to follow-up of EC deaths in rural areas. In addition, it is known that in China, EC screening covered more rural areas than urban area prior to 2012. This might lead to the contrast fallacy between rural and urban populations. However, we did not analyze survival in urban or rural areas due to the lack of original information in our study. Age is also one of the important determinants closely related to the survival of EC. Unfortunately, we did not compare the survival between age classifications due to the lack of original data for age-specific groups.

In 2015, the third global surveillance of cancer survival (CONCORD) program reported survival data from 2000 to 2014, which included 734,428 adults with EC from 60 countries (32). During the 15-years period, 5-years age-standardized survival rates ranged from 10 to 30% in most countries, which was lower than what we observed. Data from a hospital-based cancer registry between 2002 and 2014 found that the 1-, 3-, 5-, and 10-years survival rates of EC were 55.88, 26.24, 19.62, and 12.14%, respectively (15), which is also significantly lower than our results.

Limitations of our study are as follows: (1) Because the survival information is from independent studies, our findings are not representative of survival rates in China as a whole; (2) republication of survival data cannot be eliminated entirely; (3) most of the data in our study are from retrospective studies and might bias the real survival level of EC. In addition, significant publication bias was detected in both 1- and 2-years survival analyses. Positive publication bias indicates incomplete acquisition of original studies and might introduce potential fallacy to our observations.

In short, this systematic review and meta-analysis provides hospital-based survival data for EC in China. Although this study is subject to uncertain representability and obvious publication bias, it offers insight into the prognosis of EC based on in-hospital studies.

## Data Availability

All datasets generated for this study are included in the manuscript and/or the Supplementary Files.

## Author Contributions

YW and HH designed this study. ZM, XZ, and HH contributed to the literature search, review, and data extraction. MS and GD conduced statistical analyses. HH, ZM, and XZ contributed to manuscript drafting. WW and YW contributed to manuscript modification. All authors have reviewed and approved the final version of this manuscript.

### Conflict of Interest Statement

The authors declare that the research was conducted in the absence of any commercial or financial relationships that could be construed as a potential conflict of interest.
