# Supplementary material for: Survival of Esophageal Cancer in China: A Pooled Analysis on Hospital-Based Studies From 2000 to 2018
Source: Front Oncol. 2019 Jun 27;9:548. doi: 10.3389/fonc.2019.00548 (PMC6610307; doi:10.3389/fonc.2019.00548)

**[Supplementary materials]**

**Supplementary tables**

Table S1 Checklist of PRISMA Statement

| Section/Topic | Checklist Item | Reported or not |
| --- | --- | --- |
| TITLE |  |  |
| Title | Identify the report as a systematic review, meta-analysis, or both. | Y |
| ABSTRACT |  |  |
| Structured summary | Provide a structured summary including, as applicable: background; objectives; data sources; study eligibility criteria, participants, and interventions; study appraisal and synthesis methods; results; limitations; conclusions and implications of key findings; systematic review registration number. | Y |
| INTRODUCTION |  |  |
| Rationale | Describe the rationale for the review in the context of what is already known. | Y |
| Objectives | Provide an explicit statement of questions being addressed with reference to participants, interventions, comparisons, outcomes, and study design (PICOS). | Y |
| METHODS |  |  |
| Protocol and registration | Indicate if a review protocol exists, if and where it can be accessed (e.g., Web address), and, if available, provide registration information including registration number. | Y |
| Eligibility criteria | Specify study characteristics (e.g., PICOS, length of follow-up) and report characteristics (e.g., years considered, language, publication status) used as criteria for eligibility, giving rationale. | Y |
| Information sources | Describe all information sources (e.g., databases with dates of coverage, contact with study authors to identify additional studies) in the search and date last searched. | Y |
| Search | Present full electronic search strategy for at least one database, including any limits used, such that it could be repeated. | Y |
| Study selection | State the process for selecting studies (i.e., screening, eligibility, included in systematic review, and, if applicable, included in the meta-analysis). | Y |
| Data collection process | Describe method of data extraction from reports (e.g., piloted forms, independently, in duplicate) and any processes for obtaining and confirming data from investigators. | Y |
| Data items | List and define all variables for which data were sought (e.g., PICOS, funding sources) and any assumptions and simplifications made. | Y |
| Risk of bias in individual studies | Describe methods used for assessing risk of bias of individual studies (including specification of whether this was done at the study or outcome level), and how this information is to be used in any data synthesis. | Y |
| Summary measures | State the principal summary measures (e.g., risk ratio, difference in means). | Y |
| Synthesis of results | Describe the methods of handling data and combining results of studies, if done, including measures of consistency (e.g., I2) for each meta-analysis. | Y |
| Risk of bias across studies | Specify any assessment of risk of bias that may affect the cumulative evidence (e.g., publication bias, selective reporting within studies). | Y |
| Additional analyses | Describe methods of additional analyses (e.g., sensitivity or subgroup analyses, meta-regression), if done, indicating which were pre-specified. | Y |
| RESULTS |  |  |
| Study selection | Give numbers of studies screened, assessed for eligibility, and included in the review, with reasons for exclusions at each stage, ideally with a flow diagram. | Y |
| Study characteristics | For each study, present characteristics for which data were extracted (e.g., study size, PICOS, follow-up period) and provide the citations. | Y |
| Risk of bias within studies | Present data on risk of bias of each study and, if available, any outcome-level assessment (see Item 12). | Y |
| Results of individual studies | For all outcomes considered (benefits or harms), present, for each study: (a) simple summary data for each intervention group and (b) effect estimates and confidence intervals, ideally with a forest plot. | Y |
| Synthesis of results | Present results of each meta-analysis done, including confidence intervals and measures of consistency. | Y |
| Risk of bias across studies | Present results of any assessment of risk of bias across studies (see Item 15). | Y |
| Additional analysis | Give results of additional analyses, if done (e.g., sensitivity or subgroup analyses, meta-regression [see Item 16]). | Y |
| DISCUSSION |  | Y |
| Summary of evidence | Summarize the main findings including the strength of evidence for each main outcome; consider their relevance to key groups (e.g., health care providers, users, and policy makers). | Y |
| Limitations | Discuss limitations at study and outcome level (e.g., risk of bias), and at review level (e.g., incomplete retrieval of identified research, reporting bias). | Y |
| Conclusions | Provide a general interpretation of the results in the context of other evidence, and implications for future research. | Y |
| FUNDING |  |  |
| Funding | Describe sources of funding for the systematic review and other support (e.g., supply of data) | Y |

Y: the item was reported in article, N: the item was not reported.

Table S2 Search strategy

| **1. Literature published in English language** | |
| --- | --- |
| Database | PubMed; Embase; Web of Science |
| Terms | (((((China[Title/abstract/Topic])) OR (Chinese[Title/abstract/Topic])) AND (survival[Title/abstract/Topic])) AND (hospital[Title/abstract/Topic])) AND ((((((((((esophageal cancer[Title/abstract/Topic])) OR (carcinoma of esophagus[Title/abstract/Topic])) OR (esophageal carcinoma[Title/abstract/Topic])) OR (esophagus cancer[Title/abstract/Topic])) OR (cancer of esophagus[Title/abstract/Topic])) OR (eosophageal cancer[Title/abstract/Topic])) OR (carcinoma of eosophagus[Title/abstract/Topic])) OR (eosophageal carcinoma[Title/abstract/Topic])) OR (eosophagus cancer[Title/abstract/Topic])) OR (cancer of eosophagus[Title/abstract/Topic]) |
| Language | English |
| Publication dates | 2000-2018 |
| **2. Literature published in Chinese language** | |
| Database | CNKI; Wanfang |
| Terms | (食管癌[Title/abstract/Topic]) AND (生存率[Title/abstract/Topic]) AND (医院[Title/abstract/Topic]) |
| Language | Chinese |
| Publication dates | 2000-2018 |

Table S3 Scale for quality assessment based on PRISMA statement and MOOSE guideline

| **Criteria** | **Score** |
| --- | --- |
| **Representativeness of cases** |  |
| Characteristics of participants were described. | 1 |
| Consecutive/randomly selected from case population was clearly defined. | 1 |
| Eligible patients are similar to controls, in term of age, gender and other important characteristics. | 1 |
| The percentage of loss to follow-up was provided, or the reasons of loss to follow-up were mentioned. | 1 |
| **Accuracy of information** |  |
| Methods of variable measurement were offered | 1 |
| Definitions of outcome were offered. | 1 |
| **Statistical analyses** |  |
| Methods of statistical analyses were adequate to resolve research hypothesis. | 1 |
| Multivariate analyses were performed. | 1 |
| **Final question** |  |
| If there were any other important flaws in the design, the study would be not included. |  |

Table S4 Characteristics of included studies

| no | Author | Publication year | Province | Design | Age | Sample size | Male | Female | Language |
| --- | --- | --- | --- | --- | --- | --- | --- | --- | --- |
| 1 | Guo L | 2018 | Shandong | R | 55.78 | 78 | 53 | 25 | English |
| 2 | Yang D | 2018 | Beijing | R | NA | 402 | 339 | 63 | English |
| 3 | Zhang Y | 2018 | Shanghai | R | 63 | 596 | 451 | 145 | English |
| 4 | Zhang L | 2017 | Beijing | P | NA | 123 | 111 | 12 | English |
| 5 | Yang J | 2017 | Beijing | R | NA | 583 | 453 | 130 | English |
| 6 | Yang J | 2017 | Beijing | R | NA | 95 | 77 | 18 | English |
| 7 | Mu JW | 2016 | Beijing | R | 59 | 1746 | 1441 | 305 | English |
| 8 | Wang ZQ | 2016 | Sichuan | R | 60 | 258 | 208 | 50 | English |
| 9 | Zhang R | 2016 | Shandong | R | NA | 121 | 79 | 42 | English |
| 14 | Mu JW | 2015 | Beijing | R | 60 | 445 | 341 | 104 | English |
| 15 | Chen J | 2015 | Jiangsu | R | 62 | 195 | 150 | 45 | English |
| 16 | Yutong H | 2015 | Hebei | R | 60 | 820 | 546 | 274 | English |
| 24 | Wang J | 2014 | Beijing | R | NA | 1033 | 793 | 240 | English |
| 29 | Li J | 2015 | Henan | R | 59.2 | 57 | 30 | 27 | English |
| 30 | Ding J | 2013 | Jiangsu | R | 58 | 106 | 66 | 40 | English |
| 31 | Liu F | 2018 | Jiangsu | P | 68.13 | 54 | 45 | 9 | Chinese |
| 32 | Deng WJ | 2018 | Hebei | R | 64.5 | 330 | 217 | 113 | Chinese |
| 41 | Wu HR | 2018 | Anhui | R | 64.6 | 405 | 318 | 87 | Chinese |
| 42 | Wang YQ | 2018 | Hebei | R | 60 | 462 | 289 | 173 | Chinese |
| 47 | Jiang F | 2018 | Jiangsu | R | 57.85 | 127 | 90 | 37 | Chinese |
| 48 | Yang L | 2018 | Shaanxi | R | 55 | 100 | 58 | 42 | Chinese |
| 49 | Li SC | 2018 | Liaoning | R | NA | 163 | 152 | 11 | Chinese |
| 50 | Xu SB | 2018 | Anhui | R | 68 | 258 | 182 | 76 | Chinese |
| 57 | Ma R | 2018 | Tianjin | R | 61 | 454 | 381 | 73 | Chinese |
| 61 | Li CW | 2018 | Anhui | R | 63 | 253 | 203 | 50 | Chinese |
| 62 | Zhan WM | 2018 | Zhejiang | R | 57 | 105 | 70 | 35 | Chinese |
| 63 | Zhang HD | 2018 | Tianjin | R | 61 | 628 | 514 | 114 | Chinese |
| 68 | Yu Z | 2018 | Hebei | R | 62 | 212 | 148 | 64 | Chinese |
| 69 | Zhang JZ | 2018 | Hebei | R | 60.85 | 238 | 160 | 120 | Chinese |
| 73 | Shen WB | 2018 | Hebei | R | 60 | 168 | 107 | 61 | Chinese |
| 74 | Zhao YL | 2018 | Shaanxi | R | 62.06 | 144 | 83 | 61 | Chinese |
| 75 | Dai L | 2018 | Beijing | R | 59 | 167 | 140 | 27 | Chinese |
| 80 | He HJ | 2018 | Zhejiang | R | NA | 87 | 62 | 25 | Chinese |
| 81 | Li RX | 2018 | Hebei | R | 51.38 | 292 | 195 | 97 | Chinese |
| 82 | Huang Y | 2018 | Anhui | R | 77 | 110 | 87 | 23 | Chinese |
| 83 | Cheng BB | 2018 | Jiangsu | R | 51.3 | 60 | 35 | 25 | Chinese |
| 84 | GuLiMiLaMu | 2018 | Xinjiang | P | 59.89 | 110 | 65 | 45 | Chinese |
| 85 | Wu RR | 2018 | Jiangxi | P | 64 | 82 | 51 | 31 | Chinese |
| 86 | Peng HY | 2018 | Fujian | R | 72 | 57 | 43 | 14 | Chinese |
| 87 | Zhang L | 2018 | Jiangsu | P | 59.43 | 80 | 55 | 25 | Chinese |
| 88 | Sun JT | 2018 | Shandong | P | 56.5 | 120 | 66 | 54 | Chinese |
| 89 | Li M | 2018 | Henan | R | 84 | 49 | 32 | 17 | Chinese |
| 90 | Wang Z | 2018 | Henan | R | NA | 185 | 101 | 84 | Chinese |
| 91 | Ding JQ | 2018 | Fujian | P | NA | 112 | 66 | 46 | Chinese |
| 92 | Bai WW | 2018 | Hebei | R | 63 | 128 | 74 | 54 | Chinese |
| 93 | Zhu XQ | 2018 | Hunan | R | NA | 107 | 99 | 8 | Chinese |
| 94 | Cheng HZ | 2018 | Jiangsu | R | 65.44 | 470 | 337 | 133 | Chinese |
| 95 | Ren K | 2018 | Tianjin | R | NA | 144 | 120 | 24 | Chinese |
| 96 | Tu CZ | 2018 | Henan | R | 62 | 68 | 23 | 45 | Chinese |
| 97 | Li ZG | 2018 | Shanghai | R | 61 | 864 | 732 | 132 | Chinese |
| 105 | Tian Q | 2018 | Henan | R | 61 | 58 | 35 | 23 | Chinese |
| 106 | Guo XF | 2018 | Shanghai | R | 62 | 762 | 627 | 135 | Chinese |
| 107 | Xu SL | 2018 | Shandong | R | 55 | 82 | 47 | 35 | Chinese |
| 108 | Zhou YF | 2018 | Anhui | R | 56.68 | 80 | 48 | 32 | Chinese |
| 112 | Liu J | 2018 | Henan | P | 61.2 | 46 | 28 | 18 | Chinese |
| 113 | Ding BY | 2018 | Hebei | P | 59.1 | 100 | 54 | 46 | Chinese |
| 114 | Xu XJ | 2018 | Jiangsu | R | 75.93 | 41 | 32 | 9 | Chinese |
| 115 | Hou AP | 2018 | Shandong | R | 70 | 86 | 53 | 33 | Chinese |
| 116 | Wu CX | 2018 | Guangdong | R | 65 | 413 | 300 | 113 | Chinese |
| 117 | Bao EW | 2018 | Shanghai | P | NA | 103 | 76 | 27 | Chinese |
| 118 | Li ZD | 2018 | Liaoning | R | 57.4 | 92 | 92 | 0 | Chinese |
| 119 | Zhong H | 2017 | Tianjin | R | 65 | 328 | 265 | 63 | Chinese |
| 120 | Lin XH | 2017 | Anhui | R | NA | 37 | 27 | 10 | Chinese |
| 129 | Wang L | 2017 | Zhejiang | R | 58.4 | 905 | 754 | 151 | Chinese |
| 130 | Zhan BT | 2017 | Hubei | R | 60.15 | 98 | 64 | 34 | Chinese |
| 131 | Miao H | 2017 | Jiangsu | R | 76 | 64 | 43 | 21 | Chinese |
| 132 | Gao YH | 2017 | Hebei | R | 61 | 275 | 180 | 95 | Chinese |
| 136 | Fu JH | 2017 | Guangdong | R | NA | 262 | 203 | 59 | Chinese |
| 137 | Liu LH | 2017 | Hebei | R | 46.65 | 144 | 79 | 65 | Chinese |
| 138 | Chen DH | 2017 | Shandong | R | 58.9 | 60 | 35 | 25 | Chinese |
| 139 | Wang L | 2017 | Zhejiang | R | 64 | 751 | 644 | 107 | Chinese |
| 140 | Wang XA | 2017 | Sichuan | R | 52.3 | 94 | 68 | 26 | Chinese |
| 141 | Zhang NG | 2017 | Shanxi | R | 58 | 115 | 67 | 48 | Chinese |
| 142 | Li C | 2017 | Anhui | P | 65 | 11 | 10 | 1 | Chinese |
| 143 | Liang M | 2017 | Sichuan | P | 50.85 | 100 | 52 | 48 | Chinese |
| 144 | Ran G | 2017 | Zhejiang | P | 61.6 | 74 | 53 | 21 | Chinese |
| 145 | Huang WM | 2017 | Beijing | P | 54.92 | 13 | 12 | 1 | Chinese |
| 146 | Ni WJ | 2017 | Beijing | P | 60 | 218 | 184 | 34 | Chinese |
| 147 | Liu LH | 2017 | Hebei | P | 59 | 60 | 33 | 27 | Chinese |
| 148 | Li Q | 2017 | Zhejiang | P | 61 | 50 | 38 | 12 | Chinese |
| 149 | Li YN | 2017 | Qinghai | P | 61.41 | 886 | 706 | 180 | Chinese |
| 150 | Li JD | 2017 | Henan | R | 58.2 | 132 | 74 | 58 | Chinese |
| 151 | Zheng H | 2017 | Shanghai | R | 60 | 156 | 134 | 22 | Chinese |
| 152 | Liu Y | 2017 | Shanghai | P | 51.63 | 159 | 84 | 75 | Chinese |
| 153 | Gao X | 2017 | Shaanxi | R | NA | 247 | 149 | 98 | Chinese |
| 154 | Li W | 2017 | Shaanxi | R | 59.6 | 97 | 45 | 52 | Chinese |
| 155 | Sun YP | 2017 | Guizhou | P | 57.9 | 92 | 56 | 36 | Chinese |
| 156 | Su TF | 2017 | Fujian | P | 59 | 242 | 200 | 42 | Chinese |
| 157 | Wang CF | 2017 | Shandong | P | 70.2 | 102 | 61 | 41 | Chinese |
| 158 | Chen JQ | 2017 | Fujian | R | 59 | 242 | 200 | 42 | Chinese |
| 163 | Wang J | 2017 | Anhui | R | NA | 40 | NA | NA | Chinese |
| 164 | Huo XD | 2017 | Tianjin | R | 61 | 116 | 76 | 40 | Chinese |
| 165 | Zhu Q | 2017 | Jiangsu | P | 52.65 | 60 | 37 | 23 | Chinese |
| 166 | Zhao C | 2017 | Jiangsu | P | 56.1 | 40 | 25 | 15 | Chinese |
| 167 | Lan SL | 2017 | Henan | P | 52.43 | 64 | 42 | 22 | Chinese |
| 168 | Huang ZH | 2017 | Fujian | P | 49.85 | 60 | 35 | 25 | Chinese |
| 169 | Jing JW | 2017 | Henan | P | 60 | 140 | 80 | 60 | Chinese |
| 170 | Li B | 2017 | Henan | P | 68.8 | 14 | 8 | 6 | Chinese |
| 171 | Ding H | 2017 | Jiangsu | P | 65.58 | 89 | 51 | 38 | Chinese |
| 172 | Yu LH | 2017 | Henan | P | 49.6 | 40 | 24 | 16 | Chinese |
| 173 | Ming X | 2017 | Hubei | P | 48 | 96 | 59 | 37 | Chinese |
| 174 | Liu LH | 2017 | Hebei | P | 59 | 60 | 33 | 27 | Chinese |
| 175 | Wang Z | 2017 | Chongqing | P | 58.65 | 76 | 48 | 28 | Chinese |
| 176 | Wang ZD | 2017 | Shanxi | P | 58.31 | 90 | 54 | 36 | Chinese |
| 177 | Wang ZW | 2017 | Henan | P | 58.9 | 90 | 62 | 28 | Chinese |
| 178 | Xu XZ | 2017 | Henan | P | 61.49 | 62 | 41 | 21 | Chinese |
| 179 | Wang J | 2017 | Anhui | P | 66.82 | 40 | 25 | 15 | Chinese |
| 180 | Deng J | 2017 | Sichuan | R | 57.55 | 100 | 50 | 50 | Chinese |
| 181 | Wu YL | 2017 | Liaoning | P | 44 | 110 | 57 | 53 | Chinese |
| 182 | Gao X | 2017 | Shaanxi | P | NA | 247 | 149 | 98 | Chinese |
| 183 | Wang CF | 2017 | Shandong | P | 70.2 | 102 | 61 | 41 | Chinese |
| 184 | Yu LH | 2017 | Jiangsu | P | 45.25 | 90 | 55 | 35 | Chinese |
| 185 | Wang HB | 2017 | Henan | P | 64.45 | 66 | 38 | 28 | Chinese |
| 186 | Zheng GH | 2017 | Shanxi | P | 57.45 | 100 | 51 | 49 | Chinese |
| 187 | Song G | 2016 | Shandong | R | NA | 62 | 49 | 13 | Chinese |
| 194 | Xu W | 2016 | Shandong | R | 58 | 103 | 92 | 11 | Chinese |
| 195 | Li CH | 2016 | Jiangsu | R | 73.55 | 56 | 36 | 20 | Chinese |
| 196 | Pula | 2016 | Xinjiang | P | 57.1 | 120 | 70 | 50 | Chinese |
| 197 | Zhang ZW | 2016 | Jiangsu | P | 52.4 | 96 | 59 | 37 | Chinese |
| 198 | Jiang ZQ | 2016 | Guangdong | R | 52.8 | 240 | 150 | 90 | Chinese |
| 199 | Jiang ZQ | 2016 | Guangdong | R | NA | 135 | 85 | 50 | Chinese |
| 204 | Li LJ | 2016 | Chongqing | R | 62.65 | 104 | 78 | 26 | Chinese |
| 209 | Sun JY | 2016 | Neimenggu | R | 52.7 | 68 | 37 | 31 | Chinese |
| 210 | Deng WY | 2016 | Henan | R | 56 | 573 | 380 | 193 | Chinese |
| 218 | Cheng FB | 2016 | Henan | R | 44 | 100 | 56 | 46 | Chinese |
| 219 | Wang CN | 2016 | Henan | R | NA | 61 | 47 | 14 | Chinese |
| 220 | An CM | 2016 | Beijing | R | 55 | 90 | 82 | 8 | Chinese |
| 221 | Li XM | 2016 | Henan | R | 57 | 82 | 53 | 29 | Chinese |
| 222 | Huang YZ | 2016 | Henan | R | 51 | 83 | 55 | 28 | Chinese |
| 223 | Ji PJ | 2016 | Chongqing | R | 62.5 | 170 | 157 | 13 | Chinese |
| 224 | Song Q | 2016 | Sichuan | R | 82.6 | 98 | 45 | 53 | Chinese |
| 225 | Zheng H | 2016 | Guangdong | R | NA | 142 | NA | NA | Chinese |
| 226 | Zhao YH | 2016 | Beijing | R | 57.2 | 51 | 35 | 16 | Chinese |
| 227 | Cheng DX | 2016 | Liaoning | R | 54.3 | 70 | 38 | 32 | Chinese |
| 228 | Shen Q | 2016 | Beijing | R | 70.2 | 102 | 61 | 41 | Chinese |
| 229 | Chen YL | 2016 | Guangdong | R | 54.52 | 64 | 46 | 18 | Chinese |
| 230 | Lin D | 2016 | Shanghai | R | 60 | 375 | 306 | 69 | Chinese |
| 231 | Yang XD | 2016 | Shanghai | R | NA | 1021 | 1056 | 252 | Chinese |
| 232 | Liu Y | 2016 | Henan | R | 60.9 | 2558 | 1753 | 805 | Chinese |
| 240 | Chen C | 2016 | Jiangsu | R | 54.5 | 84 | 50 | 34 | Chinese |
| 241 | Li M | 2016 | Guangdong | R | NA | 269 | NA | NA | Chinese |
| 242 | Wang SE | 2016 | Henan | R | 60 | 133 | 82 | 51 | Chinese |
| 243 | Li C | 2016 | Hubei | R | 72 | 74 | 55 | 29 | Chinese |
| 244 | Guli | 2016 | Xinjiang | R | 60 | 63 | 44 | 19 | Chinese |
| 248 | Huang XQ | 2016 | Shandong | R | 56.8 | 80 | 51 | 29 | Chinese |
| 249 | Ma ZM | 2016 | Jiangsu | R | NA | 171 | 117 | 54 | Chinese |
| 250 | Li XN | 2016 | Hebei | R | 65.26 | 245 | 188 | 57 | Chinese |
| 251 | Fei LS | 2016 | Shandong | R | 51 | 98 | 66 | 32 | Chinese |
| 252 | Xu J | 2016 | Henan | R | 74 | 43 | 23 | 20 | Chinese |
| 253 | Chen WL | 2016 | Hebei | R | 57.9 | 156 | 84 | 72 | Chinese |
| 254 | Guo JF | 2016 | Shaanxi | P | NA | 60 | NA | NA | Chinese |
| 255 | Niu SH | 2016 | Liaoning | R | 62 | 93 | 80 | 13 | Chinese |
| 256 | Li DJ | 2016 | Anhui | R | 71 | 88 | 58 | 30 | Chinese |
| 257 | Bai WW | 2016 | Hebei | R | 63 | 63 | 31 | 32 | Chinese |
| 260 | Yang JM | 2016 | Tianjin | R | 62 | 136 | 112 | 24 | Chinese |
| 267 | Mao AY | 2016 | Guangdong | R | 56.3 | 189 | 113 | 76 | Chinese |
| 268 | Deng WY | 2016 | Beijing | R | 56 | 103 | 91 | 12 | Chinese |
| 269 | Xu G | 2016 | Jiangsu | P | 58 | 48 | 31 | 17 | Chinese |
| 270 | Chen L | 2016 | Jiangsu | R | NA | 90 | 58 | 32 | Chinese |
| 271 | Yu SF | 2016 | Beijing | R | NA | 286 | 226 | 60 | Chinese |
| 272 | Kong M | 2016 | Zhejiang | R | 61.3 | 622 | 431 | 205 | Chinese |
| 273 | Wang DM | 2016 | Chongqing | R | 60 | 256 | 165 | 91 | Chinese |
| 274 | Yong BQ | 2016 | Zhejiang | R | 68 | 97 | 83 | 14 | Chinese |
| 275 | Zhao QW | 2015 | Shanxi | R | 47 | 156 | 98 | 58 | Chinese |
| 276 | Zhou C | 2015 | Jiangsu | R | 71 | 112 | 71 | 41 | Chinese |
| 277 | Xu ZN | 2015 | Anhui | R | 59.4 | 76 | 43 | 33 | Chinese |
| 278 | Shen W | 2015 | Henan | R | 60 | 62 | 40 | 22 | Chinese |
| 279 | MinBB | 2015 | Hubei | R | 51.32 | 44 | 31 | 13 | Chinese |
| 280 | Wei GB | 2015 | Guangdong | P | 58 | 178 | 124 | 54 | Chinese |
| 281 | Liu GJ | 2015 | Hebei | R | 54.4 | 17 | 13 | 4 | Chinese |
| 282 | Liu Y | 2015 | Henan | R | 66 | 191 | 116 | 75 | Chinese |
| 287 | Xu XX | 2015 | Henan | R | 61.03 | 2724 | 1873 | 851 | Chinese |
| 288 | Li LL | 2015 | Henan | R | 62 | 137 | 113 | 24 | Chinese |
| 289 | Wang S | 2015 | Jiangsu | R | 54.5 | 60 | 35 | 25 | Chinese |
| 290 | Cao YK | 2015 | Hebei | R | NA | 158 | 87 | 71 | Chinese |
| 295 | Wang W | 2015 | Xinjiang | R | 40.85 | 60 | 35 | 25 | Chinese |
| 296 | Liu Y | 2015 | Henan | R | 66 | 191 | 116 | 75 | Chinese |
| 301 | Wang M | 2015 | Guangdong | R | 54 | 362 | 253 | 109 | Chinese |
| 302 | Wang ZH | 2015 | Henan | P | NA | 55 | 30 | 25 | Chinese |
| 303 | Wang LQ | 2015 | Jilin | R | 54.8 | 86 | 45 | 41 | Chinese |
| 304 | Wu SF | 2015 | Hubei | R | 70.5 | 92 | 62 | 30 | Chinese |
| 305 | Ma YF | 2015 | Beijing | R | 52.3 | 240 | 186 | 54 | Chinese |
| 306 | Yuan Y | 2015 | Jiangsu | R | 60.9 | 60 | 39 | 21 | Chinese |
| 307 | Wang YD | 2016 | Hebei | R | 70 | 34 | 23 | 11 | Chinese |
| 308 | Zhang TJ | 2016 | Henan | R | 61.4 | 241 | 147 | 94 | Chinese |
| 309 | He J | 2002 | Shanghai | R | 53 | 86 | 77 | 9 | Chinese |
| 310 | Lei DP | 2002 | Shandong | R | 58.8 | 33 | 31 | 2 | Chinese |
| 311 | Zhou YF | 2003 | Henan | R | NA | 3997 | NA | NA | Chinese |
| 312 | Hong M | 2004 | Fujian | R | 55 | 101 | 89 | 22 | Chinese |
| 313 | Xu SM | 2005 | Shanghai | R | 65.8 | 12 | 10 | 2 | Chinese |
| 314 | Li R | 2005 | Henan | P | 55 | 128 | 96 | 32 | Chinese |
| 315 | Cai HX | 2006 | Fujian | R | NA | 535 | NA | NA | Chinese |
| 316 | Wang X | 2006 | Hebei | R | 58 | 102 | 69 | 33 | Chinese |
| 317 | Li CH | 2006 | Shanxi | R | 61 | 102 | NA | NA | Chinese |
| 318 | Wang CL | 2007 | Shanxi | R | 57.16 | 282 | 185 | 97 | Chinese |
| 319 | Wu XA | 2007 | Henan | P | 57 | 48 | 32 | 16 | Chinese |
| 320 | Xu JH | 2008 | Fujian | R | 53.4 | 86 | 51 | 35 | Chinese |
| 321 | Wang WG | 2008 | Henan | R | 51.8 | 82 | 67 | 15 | Chinese |
| 322 | Wang CL | 2008 | Shanxi | R | 57.01 | 1140 | 742 | 398 | Chinese |
| 323 | Mu JW | 2008 | Beijing | R | NA | 34 | 29 | 5 | Chinese |
| 324 | Huang JY | 2009 | Henan | R | 53 | 60 | 35 | 25 | Chinese |
| 325 | Jiang J | 2009 | National | R | 67 | 132 | 104 | 28 | Chinese |
| 327 | Liu JW | 2009 | Henan | P | 56.42 | 147 | 97 | 50 | Chinese |
| 328 | Lv JM | 2009 | Beijing | R | NA | 126 | 99 | 27 | Chinese |
| 329 | Yu YJ | 2009 | Shandong | R | 62.9 | 66 | 46 | 20 | Chinese |
| 330 | Wu ZY | 2009 | Guangdong | R | 52 | 411 | 300 | 111 | Chinese |
| 331 | Chen XY | 2009 | Henan | R | 60.9 | 3169 | 2115 | 1054 | Chinese |
| 332 | Huo XD | 2010 | Xinjiang | R | 61.03 | 339 | 249 | 90 | Chinese |
| 333 | Lv YL | 2010 | Hebei | R | NA | 97 | 72 | 25 | Chinese |
| 334 | Song L | 2010 | Shandong | R | 46 | 165 | 125 | 40 | Chinese |
| 338 | Hao JL | 2010 | Tianjin | R | 60.2 | 196 | 132 | 64 | Chinese |
| 339 | Long S | 2010 | Guangdong | R | 51.6 | 70 | 43 | 27 | Chinese |
| 340 | Gao M | 2010 | Hebei | R | 59 | 90 | 67 | 23 | Chinese |
| 341 | Du FF | 2010 | Tianjin | R | NA | 89 | 68 | 21 | Chinese |
| 342 | Lu QG | 2010 | Jiangsu | R | 64.4 | 200 | 120 | 80 | Chinese |
| 343 | Deng GM | 2011 | Guangdong | R | 72 | 62 | 43 | 19 | Chinese |
| 346 | Zhang YD | 2011 | Liaoning | R | 56.3 | 80 | 62 | 18 | Chinese |
| 347 | Cha TZ | 2011 | Jiangsu | R | 59.1 | 341 | 271 | 70 | Chinese |
| 353 | Wang YX | 2011 | Hebei | R | 64.5 | 208 | 141 | 67 | Chinese |
| 354 | Song L | 2011 | Shandong | R | 58.8 | 184 | 143 | 41 | Chinese |
| 358 | Xu YP | 2011 | Zhejiang | R | 56 | 196 | 171 | 25 | Chinese |
| 359 | Lv J | 2011 | Jiangsu | P | NA | 233 | 145 | 88 | Chinese |
| 360 | Feng J | 2011 | Shanghai | R | 58.2 | 204 | 168 | 36 | Chinese |
| 361 | Ma SH | 2011 | Beijing | R | 60 | 493 | NA | NA | Chinese |
| 362 | Li GH | 2012 | Anhui | R | 64 | 33 | 25 | 8 | Chinese |
| 363 | Chen XP | 2012 | Sichuan | R | 55.4 | 56 | 31 | 25 | Chinese |
| 364 | Zhang WC | 2012 | Beijing | R | 57 | 251 | 212 | 39 | Chinese |
| 368 | Xing J | 2012 | Hunan | R | 53.2 | 134 | 90 | 44 | Chinese |
| 369 | Zhang XB | 2012 | Zhejiang | R | 73 | 33 | 25 | 8 | Chinese |
| 370 | Ma SH | 2012 | Beijing | R | 61 | 521 | 399 | 122 | Chinese |
| 371 | Yin HT | 2012 | Heilongjiang | R | 79 | 70 | 61 | 9 | Chinese |
| 372 | Dong GH | 2012 | Jiangsu | R | 52.6 | 36 | 26 | 10 | Chinese |
| 373 | Zhu QS | 2012 | Henan | R | 61 | 323 | NA | NA | Chinese |
| 374 | Xu Q | 2012 | Hebei | R | 60 | 607 | 413 | 194 | Chinese |
| 375 | Liang XQ | 2012 | Shanxi | R | 59 | 736 | 438 | 298 | Chinese |
| 376 | Zhu SC | 2012 | Hebei | R | 60 | 618 | 424 | 194 | Chinese |
| 377 | Aheli | 2012 | Xinjiang | R | NA | 249 | 182 | 67 | Chinese |
| 378 | Chen CZ | 2012 | Guangdong | R | 63 | 236 | 179 | 57 | Chinese |
| 379 | An HY | 2012 | Jiangsu | R | 59.5 | 61 | 33 | 28 | Chinese |
| 380 | Yang H | 2012 | Shanghai | R | NA | 123 | 100 | 23 | Chinese |
| 381 | Zeng YD | 2012 | Hunan | P | NA | 60 | 32 | 28 | Chinese |
| 382 | Fan HY | 2012 | Hebei | R | 59.3 | 55 | 34 | 21 | Chinese |
| 386 | Tao H | 2012 | Jiangsu | R | 60 | 49 | 37 | 12 | Chinese |
| 387 | Mao YS | 2012 | Beijing | R | 58.8 | 559 | 282 | 277 | Chinese |
| 388 | Du ZS | 2012 | Guangdong | R | 57.59 | 239 | 180 | 59 | Chinese |
| 389 | Zhu SM | 2012 | Shanghai | P | NA | 60 | 39 | 21 | Chinese |
| 390 | Chen HZ | 2013 | Guangdong | P | NA | 86 | 54 | 32 | Chinese |
| 391 | Wei JB | 2013 | Guangdong | R | 70 | 100 | 68 | 32 | Chinese |
| 392 | Mao ZM | 2013 | Jiangsu | P | 65 | 303 | 182 | 121 | Chinese |
| 393 | Lan YL | 2013 | Beijing | R | 62 | 34 | 32 | 2 | Chinese |
| 394 | Liu FX | 2013 | Henan | R | NA | 70 | 53 | 17 | Chinese |
| 395 | Li JP | 2013 | Shanghai | R | NA | 827 | 619 | 208 | Chinese |
| 396 | Feng LJ | 2013 | Henan | R | 53.7 | 48 | 41 | 7 | Chinese |
| 397 | Zhang S | 2013 | Hebei | R | NA | 160 | 82 | 78 | Chinese |
| 398 | Li DJ | 2013 | Anhui | R | 64.6 | 68 | 46 | 22 | Chinese |
| 399 | Cao F | 2013 | Hebei | R | NA | 182 | 118 | 64 | Chinese |
| 400 | Gao L | 2013 | Henan | P | 79 | 62 | 42 | 20 | Chinese |
| 401 | Wang GM | 2013 | Anhui | P | 69.5 | 74 | 47 | 27 | Chinese |
| 402 | Ji FZ | 2013 | Jiangsu | P | 57.5 | 160 | 125 | 35 | Chinese |
| 403 | Wu TY | 2013 | Shanxi | R | 62 | 118 | 78 | 50 | Chinese |
| 404 | Cai YJ | 2013 | Fujian | R | 54.48 | 197 | 130 | 67 | Chinese |
| 405 | Zang RK | 2013 | Shandong | P | 59.4 | 73 | 56 | 17 | Chinese |
| 406 | Ayiguli | 2014 | Xinjiang | R | 67 | 58 | 40 | 18 | Chinese |
| 407 | Gan ZH | 2014 | Guangxi | R | 52 | 60 | 38 | 22 | Chinese |
| 411 | Li XM | 2014 | Henan | P | 54.2 | 3160 | 2165 | 995 | Chinese |
| 412 | Su N | 2014 | Hebei | R | 64 | 72 | 43 | 29 | Chinese |
| 413 | Ma Z | 2014 | Tianjin | R | 62 | 126 | 109 | 17 | Chinese |
| 414 | Shao MW | 2014 | Jiangsu | R | 60.7 | 686 | 501 | 185 | Chinese |
| 415 | Li Y | 2014 | Shanxi | P | 53.5 | 56 | 27 | 29 | Chinese |
| 416 | Wang WH | 2014 | Shanghai | P | 60.6 | 112 | 69 | 43 | Chinese |
| 417 | Li L | 2014 | Hainan | P | 55.8 | 120 | 68 | 52 | Chinese |
| 418 | Liu MX | 2014 | Henan | R | 61 | 2226 | 1336 | 890 | Chinese |
| 419 | Cai JD | 2014 | Hebei | P | 59 | 99 | 67 | 32 | Chinese |
| 420 | Hu J | 2014 | Sichuan | P | 65.4 | 80 | 51 | 29 | Chinese |
| 421 | Wang XX | 2014 | Hebei | P | 44 | 80 | 41 | 39 | Chinese |
| 422 | Tian DL | 2014 | Jiangsu | P | 56.6 | 78 | 51 | 27 | Chinese |
| 423 | Peng SZ | 2014 | Guangdong | P | 66.7 | 45 | 29 | 15 | Chinese |
| 424 | Jiang C | 2014 | Tianjin | R | 62 | 29 | 25 | 4 | Chinese |
| 431 | Li Y | 2014 | Shanxi | P | 57 | 64 | 31 | 33 | Chinese |
| 432 | Jiang C | 2014 | Tianjin | R | 62 | 33 | 27 | 6 | Chinese |
| 436 | Yan JM | 2014 | Zhejiang | P | 44 | 160 | 82 | 78 | Chinese |
| 437 | Chen JQ | 2014 | Fujian | R | NA | 547 | 406 | 141 | Chinese |
| 438 | Cao ZA | 2014 | Shanghai | R | 67.2 | 137 | 96 | 41 | Chinese |
| 439 | Rao XH | 2014 | Guangdong | P | 62.7 | 228 | 138 | 90 | Chinese |
| 440 | Du Y | 2014 | Hebei | P | 66.4 | 126 | 73 | 53 | Chinese |
| 441 | Tian P | 2014 | Shandong | R | 58.8 | 184 | 143 | 41 | Chinese |
| 442 | Ma XM | 2014 | Hebei | P | NA | 80 | 49 | 31 | Chinese |
| 443 | Pang XY | 2014 | Guangdong | P | NA | 91 | 62 | 29 | Chinese |
| 444 | Liu SY | 2014 | Fujian | R | 56 | 1551 | 1180 | 381 | Chinese |
| 445 | Liu LX | 2014 | Henan | P | 65.9 | 83 | 51 | 32 | Chinese |
| 446 | Dong X | 2014 | Shandong | P | 62 | 248 | 184 | 64 | Chinese |
| 447 | Guo XR | 2014 | Shanxi | P | 63.42 | 641 | 427 | 214 | Chinese |
| 448 | Wang CW | 2014 | Shandong | P | NA | 63 | 59 | 4 | Chinese |
| 449 | Hao YK | 2014 | Hebei | R | 62.5 | 36 | 21 | 15 | Chinese |
| 450 | Ge XG | 2014 | Jiangsu | R | 57.12 | 269 | 148 | 121 | Chinese |
| 451 | Li SH | 2014 | Shandong | R | NA | 75 | 56 | 19 | Chinese |
| 452 | Liu X | 2014 | Beijing | R | 62 | 290 | 237 | 53 | Chinese |
| 453 | Wang QL | 2014 | Henan | P | 46.17 | 151 | 97 | 54 | Chinese |
| 454 | Pan XH | 2014 | Guangdong | P | 51.2 | 80 | 44 | 36 | Chinese |
| 455 | Wu Q | 2014 | Hunan | P | 58.9 | 92 | 52 | 40 | Chinese |
| 456 | Liu X | 2014 | Beijing | R | NA | 358 | 277 | 81 | Chinese |
| 457 | Lu ZB | 2014 | Jiangxi | P | 46.85 | 120 | 67 | 53 | Chinese |
| 458 | Mei K | 2014 | Hunan | P | 44.5 | 146 | 94 | 52 | Chinese |
| 459 | Wang WG | 2014 | Jiangsu | R | NA | 1376 | 1223 | 153 | Chinese |
| 460 | Jiang W | 2014 | Shandong | P | 54 | 100 | 61 | 39 | Chinese |
| 461 | Yu XY | 2014 | Fujian | P | 59 | 33 | 26 | 7 | Chinese |
| 462 | Wu B | 2014 | Zhejiang | P | NA | 70 | 49 | 21 | Chinese |
| 463 | Ling JH | 2014 | Sichuan | P | NA | 80 | 46 | 34 | Chinese |
| 464 | Ma K | 2014 | Sichuan | P | 57.6 | 127 | 92 | 35 | Chinese |
| 465 | Zhang SQ | 2014 | Guangdong | P | 53.6 | 65 | 39 | 26 | Chinese |
| 466 | Zhang AD | 2015 | Hebei | R | 66 | 1349 | 837 | 512 | Chinese |
| 467 | Rao ZP | 2015 | Guangdong | P | 45.3 | 80 | 43 | 37 | Chinese |
| 468 | Feng YJ | 2015 | Sichuan | P | NA | 76 | 50 | 26 | Chinese |
| 469 | Huang HB | 2015 | Hainan | P | 57.35 | 126 | 90 | 36 | Chinese |
| 473 | Wang H | 2015 | Shandong | P | 64.3 | 136 | 79 | 57 | Chinese |
| 474 | Zhou JH | 2015 | Hubei | P | NA | 207 | 135 | 72 | Chinese |
| 475 | Gao JW | 2015 | Guangdong | P | 56.3 | 50 | 37 | 13 | Chinese |
| 476 | Yang SP | 2015 | Jiangsu | P | NA | 66 | 37 | 29 | Chinese |
| 477 | Lv B | 2015 | Shanghai | R | 74 | 53 | NA | NA | Chinese |
| 478 | Kong Y | 2015 | Hebei | R | 65 | 170 | 108 | 62 | Chinese |
| 479 | Zhu LY | 2015 | Hebei | P | 45 | 80 | 53 | 27 | Chinese |
| 480 | Zhong HH | 2015 | Guangdong | P | 53.4 | 80 | 52 | 28 | Chinese |
| 481 | Liu Z | 2015 | Sichuan | P | NA | 430 | 253 | 177 | Chinese |
| 482 | Gao L | 2015 | Henan | R | NA | 41 | 30 | 11 | Chinese |
| 483 | Wang F | 2015 | Tianjin | R | 63 | 135 | 108 | 27 | Chinese |
| 487 | Liu YS | 2015 | Anhui | P | NA | 86 | 71 | 15 | Chinese |
| 488 | Rao XH | 2015 | Guangdong | R | 58 | 200 | 144 | 56 | Chinese |
| 489 | Gao QF | 2015 | Shandong | P | 57.35 | 80 | 48 | 32 | Chinese |
| 490 | Yang J | 2015 | Jiangsu | R | NA | 124 | 90 | 34 | Chinese |
| 491 | Li JD | 2015 | Henan | R | 59.2 | 57 | 30 | 27 | Chinese |
| 492 | Chen L | 2017 | Beijing | R | 58.9 | 136 | 116 | 20 | Chinese |

P, prospective study; R, retrospective study; NA, not available

Table S5 Temporal trends of survival for esophageal cancer from 2000 to 2018

| Survival | 2000-2005 | |  | 2006-2010 | |  | 2011-2015 | |  | 2016-2018 | |  | Overall | |
| --- | --- | --- | --- | --- | --- | --- | --- | --- | --- | --- | --- | --- | --- | --- |
|  | N | SR (%) |  | N | SR (%) |  | N | SR (%) |  | N | SR (%) |  | N | SR (%) |
| One-year | 5 | 69.9 (61.2-78.7) |  | 17 | 70.7 (65.3-76) |  | 105 | 73.3 (70.9-75.8) |  | 105 | 75.7 (73.4-78) |  | 231 | 74.1 (72.6-75.7) |
| Two-year | 1 | 38 (30.2-45.8) ^a^ |  | 6 | 28.7 (9.9-47.4) ^a^ |  | 58 | 47.1 (40.3-53.9) ^a^ |  | 60 | 53.1 (46.3-59.9) ^a^ |  | 125 | 49.0 (44.2-53.8) ^a^ |
| Three-year | 5 | 36.7 (29.4-44.1) ^a^ |  | 16 | 32 (24.6-39.4) ^a^ |  | 93 | 43.3 (38.2-48.3) ^a^ |  | 97 | 51.4 (46.4-56.4) ^a^ |  | 210 | 46.0 (42.6-49.5) ^a^ |
| Five-year | - | NA |  | - | NA |  | 16 | 36.8 (21.4-52.1) ^a^ |  | 57 | 40.9 (33.9-48) ^a^ |  | 72 | 40.1 (33.7-46.4) ^a^ |

SR, survival rate; CI, confidence interval; N, number of included studies; Between-group comparisons: a, *P*<0.05 compared with one-year survival rate.

Table S6 Temporal trends of survival for esophageal cancer among men from 2000 to 2018

| Survival | 2000-2005 | |  | 2006-2010 | |  | 2011-2015 | |  | 2016-2018 | |  | Overall | |
| --- | --- | --- | --- | --- | --- | --- | --- | --- | --- | --- | --- | --- | --- | --- |
|  | N | SR (%) |  | N | SR (%) |  | N | SR (%) |  | N | SR (%) |  | N | SR (%) |
| One-year | - | NA |  | - | NA |  | 9 | 64.3 (52.7-75.9) |  | 10 | 75.6 (66.0-85.2) |  | 19 | 70.6 (63.9-77.2) |
| Two-year | - | NA |  | - | NA |  | 3 | 28.1 (17.6-38.6) ^a^ |  | 3 | 39.2 (25.5-52.8) ^a^ |  | 6 | 33.9 (25.7-42.0) ^a^ |
| Three-year | - | NA |  | - | NA |  | 10 | 42.2 (33.0-51.3) ^a^ |  | 13 | 45.2(33.7-56.8) ^a^ |  | 23 | 43.9 (37.2-50.6) ^a^ |
| Five-year | - | NA |  | 2 | 41.4 (33.6-49.2) |  | 10 | 36.5 (32.5-40.5) ^a^ |  | 15 | 40.5(32.3-48.6) ^a^ |  | 27 | 38.8 (34.2-43.4) ^a^ |

SR, survival rate; CI, confidence interval; N, number of included studies; Between-group comparisons: a, *P*<0.05 compared with one-year survival rate.

Table S7 Temporal trends of survival for esophageal cancer among women from 2000 to 2018

| Survival | 2000-2005 | |  | 2006-2010 | |  | 2011-2015 | |  | 2016-2018 | |  | Overall | |
| --- | --- | --- | --- | --- | --- | --- | --- | --- | --- | --- | --- | --- | --- | --- |
|  | N | SR (%) |  | N | SR (%) |  | N | SR (%) |  | N | SR (%) |  | N | SR (%) |
| One-year | - | NA |  | - | NA |  | 9 | 60.9 (39.8-82.0) |  | 10 | 76.9 (68.6-85.2) |  | 19 | 69.8 (58.4-81.2) |
| Two-year | - | NA |  | - | NA |  | 2 | 31.3 (15.3-47.2) |  | 3 | 41.7 (34.8-48.6) ^a^ |  | 5 | 40.0 (33.7-46.4) ^a^ |
| Three-year | - | NA |  | - | NA |  | 10 | 43.3 (29.6-57.0) |  | 12 | 55.6(44.8-66.3) ^a^ |  | 22 | 50.0 (39.9-60.1) |
| Five-year | - | NA |  | 2 | 38.3 (23.7-52.9) |  | 10 | 40.5 (28.6-52.4) |  | 15 | 46.1(37.5-54.8) ^a^ |  | 27 | 43.4 (35.9-50.9) ^a^ |

SR, survival rate; CI, confidence interval; N, number of included studies; Between-group comparisons: a, *P*<0.05 compared with one-year survival rate.

Table S8 Pooled survival and 95% CI of esophageal cancer in prospective and retrospective studies

| Survival | Retrospective | |  | Prospective | |  | Overall | |
| --- | --- | --- | --- | --- | --- | --- | --- | --- |
|  | N | SR (%) |  | N | SR (%) |  | N | SR (%) |
| One-year | 147 | 75.3 (73.5-77.2) |  | 84 | 71.9 (68.6-75.2) |  | 231 | 74.1 (72.6-75.7) |
| Two-year | 74 | 49.6 (43.3-55.9) ^a^ |  | 51 | 48.1 (41.5-54.8) ^a^ |  | 125 | 49.0 (44.2-53.8) ^a^ |
| Three-year | 156 | 45.5 (41.4-49.6) ^a^ |  | 54 | 47.6 (42.8-52.3) ^a^ |  | 210 | 46.0 (42.6-49.5) ^a^ |
| Five-year | 65 | 39.8 (33.0-46.7) ^a^ |  | 7 | 42.6 (34.7-50.4) ^a^ |  | 72 | 40.1 (33.7-46.4) ^a^ |

SR, survival rate; CI, confidence interval; N, number of included studies; Between-group comparisons: a, *P*<0.05 compared with one-year survival rate.

**Supplementary figures**

Figure S1 Sensitivity analysis for meta-analysis of one-year survival


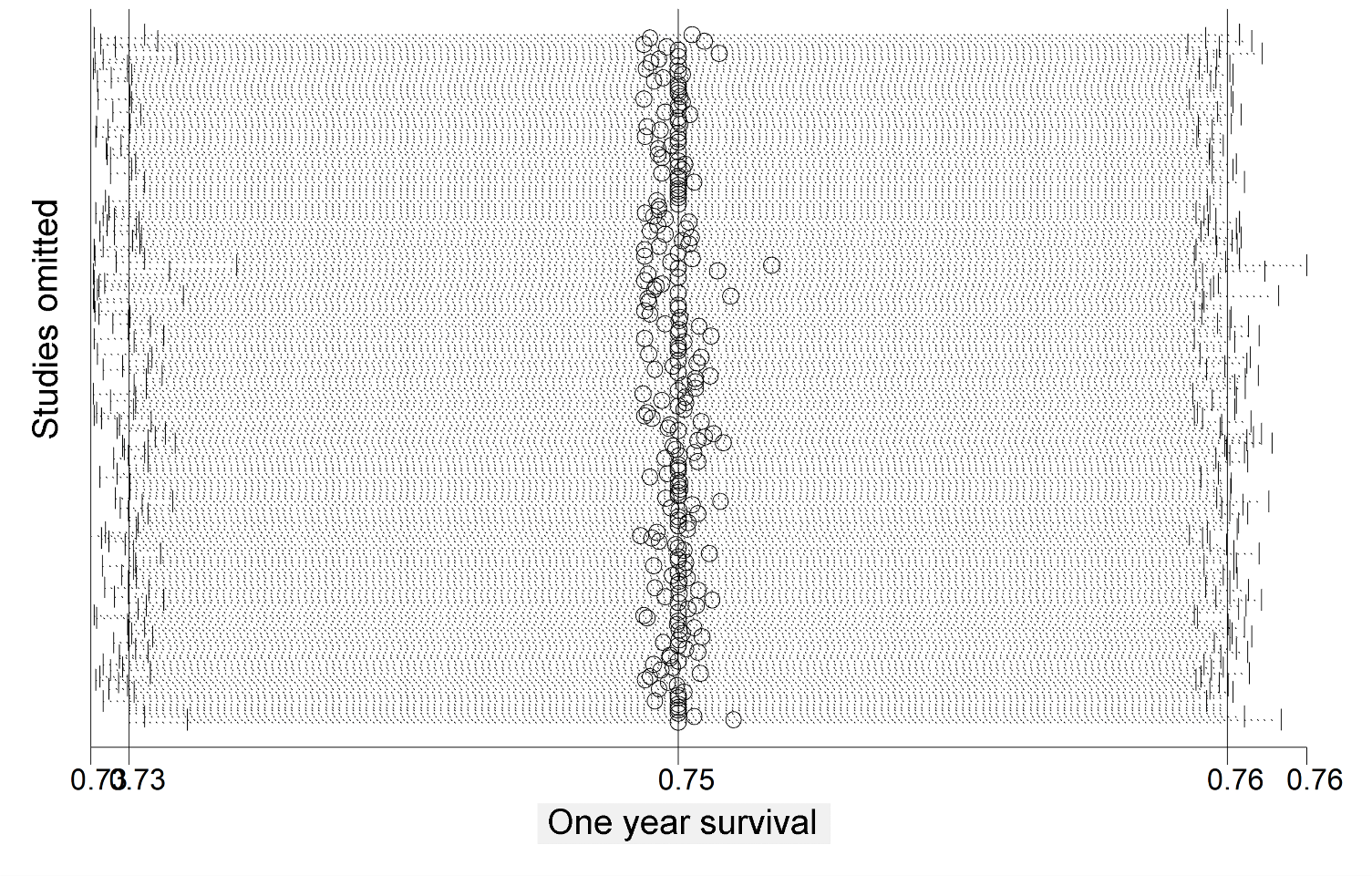


Figure S2 Sensitivity analysis for meta-analysis of two-year survival


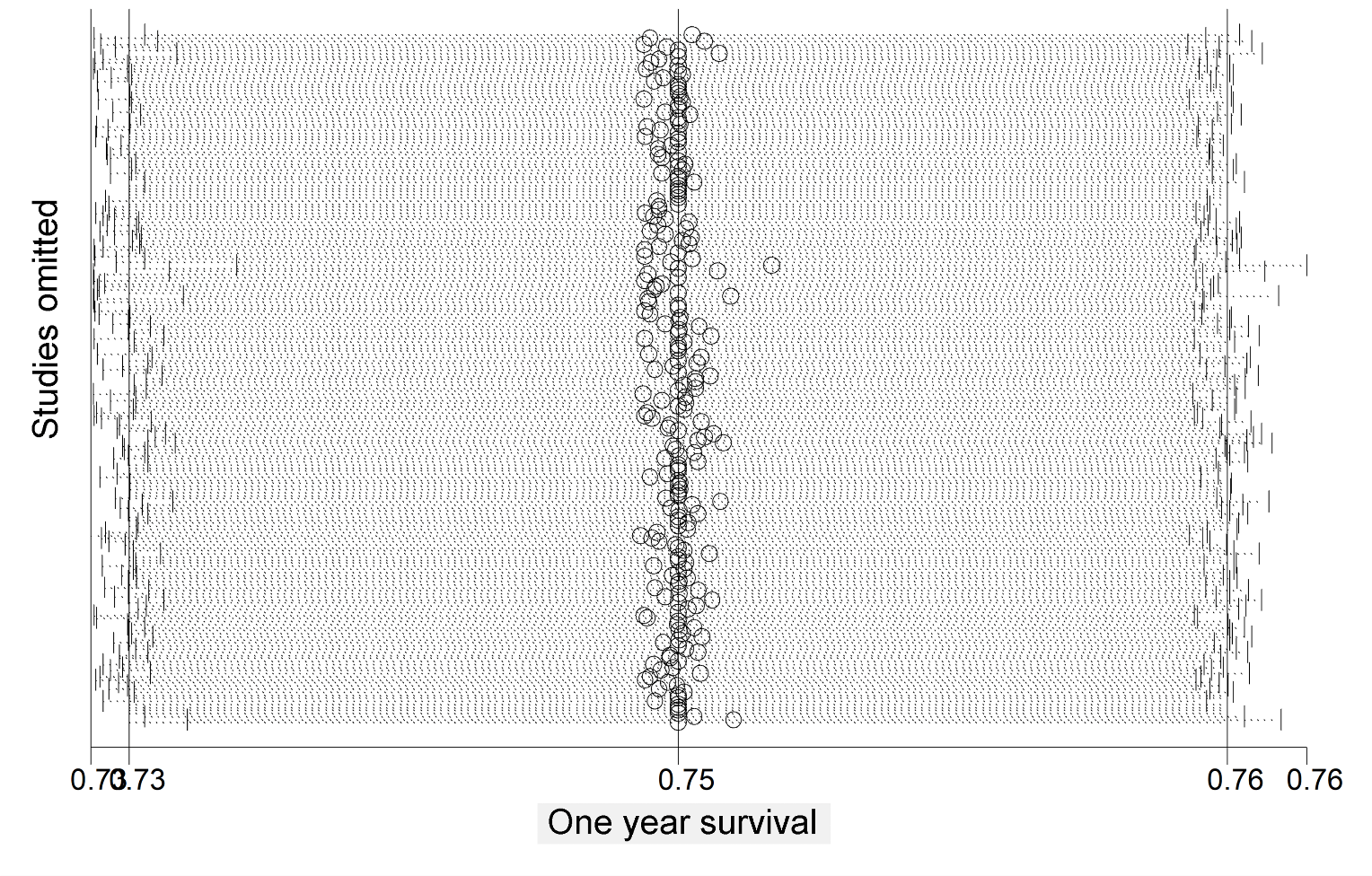


Figure S3 Sensitivity analysis for meta-analysis of three-year survival


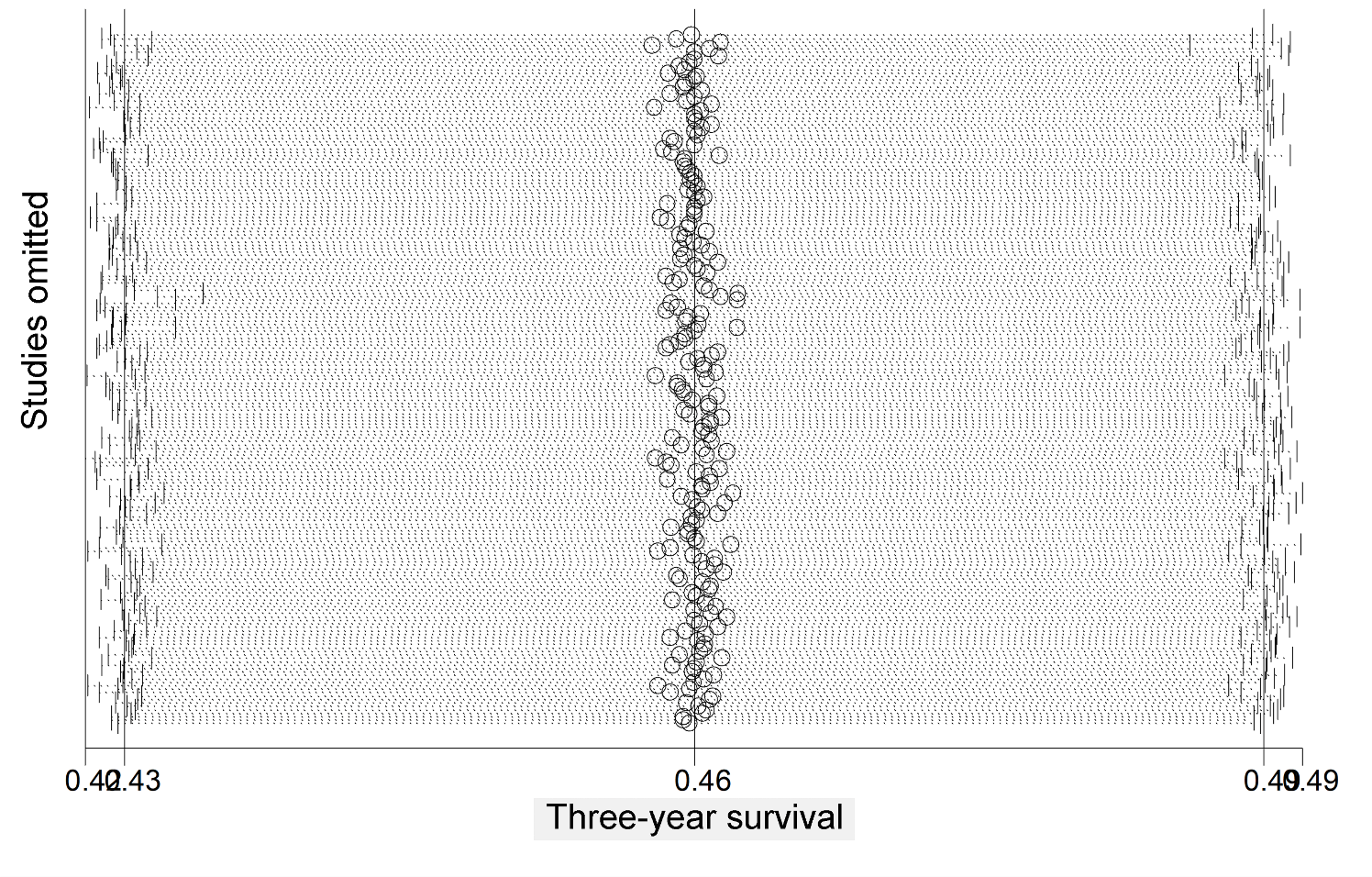


Figure S4 Sensitivity analysis for meta-analysis of five-year survival


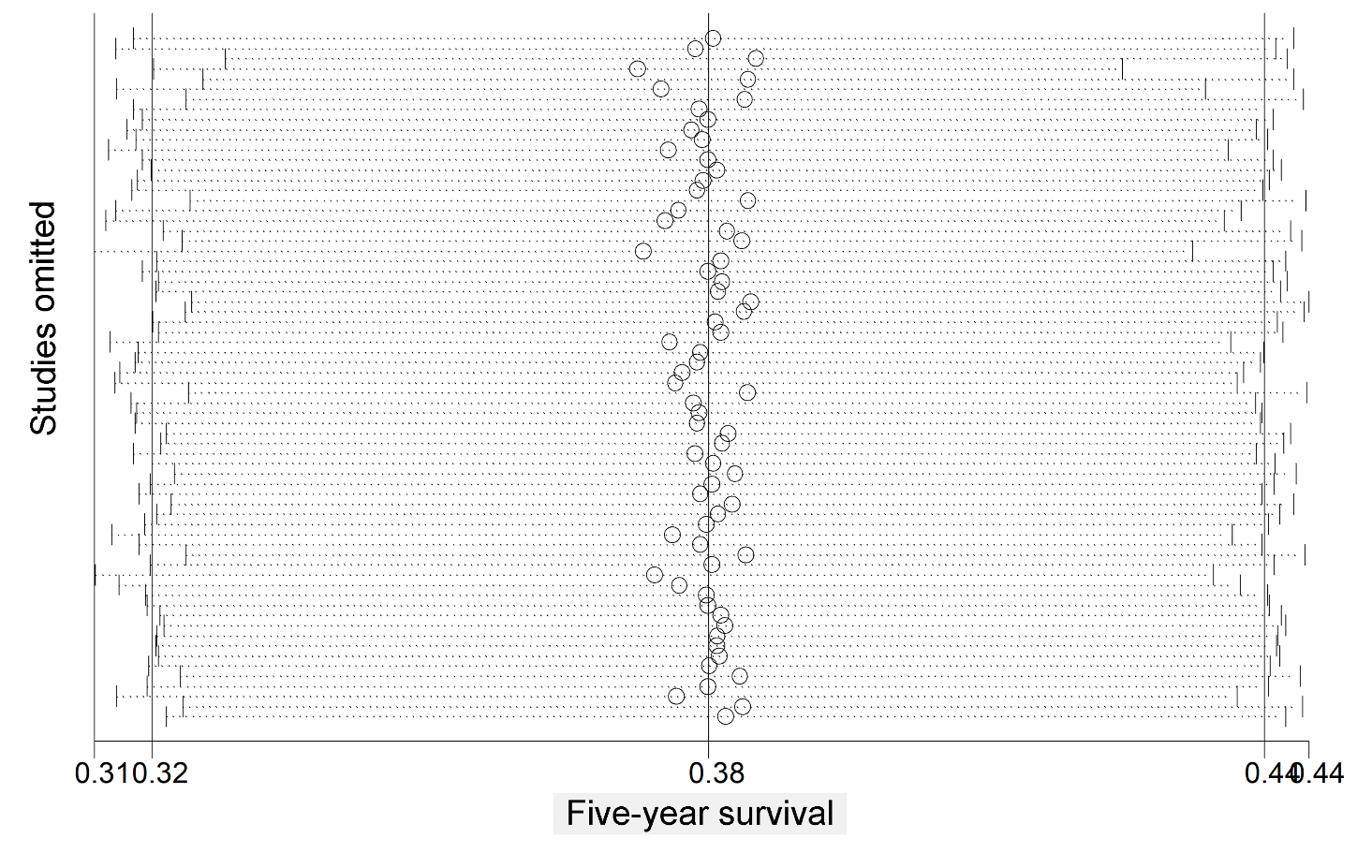

Supplement: Supplementary file 1 [file Data_Sheet_1.docx]
